# Supplementary material for: A new species of Leptobrachium (Anura, Megophryidae) from western Thailand
Source: PeerJ. 2018 Aug 31;6:e5584. doi: 10.7717/peerj.5584 (PMC6120444; doi:10.7717/peerj.5584)
Supplement: Table S1 — Sample IDs correspond to those shown in Fig. 2. GenBank AN –GenBank Accession Number. [file peerj-06-5584-s001.docx]

**SUPPLEMENTARY TABLE 1.**

| **Sample ID** | **Species** | **GenBank AN** | **Voucher** | **Locality** |
| --- | --- | --- | --- | --- |
|  | **Ingroup** |  |  |  |
| **1** | *L. abbotti* | AB646398 | KUHE39294 | Malaysia, Sabah, Kinabalu Mt., Poring |
| **2** | *L. ailaonicum echinatum* | AB719257 | MNHN1999.5657 | Vietnam, Lao Cai Prov., Sa Pa |
| **3** | *L. ailaonicum ailaonicum* | EF544224 | IZCASH30027 | China, Yunnan Prov., Ailaoshan Mt. |
| **4** | *L. banae* | EF544229 | ROM32200 | Vietnam, Gia Lai Prov., Krong Pa |
| **5** | *L. boringii* | EF544207 | IZCASH30021 | China, Sichuan Prov., Emeishan Mt. |
| **6** | *L. buchardi* | HQ709353 | FMNH258086 | Laos, Champasak Prov., Boloven Highlands, Paksong Distr. |
| **7** | *L.* cf. *chapaense* | DQ283052 | AMNHA163791 | Vietnam, Ha Giang Prov., Vi Xuyen, Cao Bo |
| **8** | *L. cf. guangxiense* | EF544232 | ROM32176 | Vietnam, Vinh Phuc Prov., Tam Dao |
| **9** | *L.* cf. *montanum* | GQ995543 | AH196 | Malaysia, Sarawak, Mulu N.P. |
| **10** | *L.* cf. *montanum* | AB646406 | KUHE08073 | Malaysia, Sabah, Tampaluri |
| **11** | *L. chapaense* | KR018126 | AMSR171623 | Vietnam, Lao Cai Prov., Sa Pa |
| **12** | *L. guangxiense* | JX467672 | 200807003 | China, Guangxi Prov., Shiwanshan Mt. |
| **13** | *L. gunungense* | AB646405 | KUHE39377 | Malaysia, Sabah, Kinabalu Mt., Sungai Carson |
| **14** | *L. hainanense* | AB530447 | KUHEUNLL68 | China, Hainan Prov., Wuzhishan Mt. |
| **15** | *L. hasseltii* | AB646408 | KUHE44535 | Indonesia, Java, Central Java, Ungaran Mt. |
| **16** | *L. hendricksoni* | AB530411 | KUHE15336 | Malaysia, Penang |
| **17** | *L. hendricksoni* | AB530417 | KUHE15680 | Malaysia, Kuala Lumpur |
| **18** | *L. huashen* | AB530443 | ROM41243 | China, Yunnan Prov., Simao |
| **19** | *L. huashen* | AB530442 | KUHEUNtissueL57 | Thailand, Chiang Mai Prov., Doi Angkang |
| **20** | *L. huashen* | AB530444 | KUHE19122 | Thailand, Chiang Mai Prov., Doi Intanon, Ban Khun Klang |
| **21** | *L. ingeri* | AB719253 | MZBAmp11791 | Indonesia, Belitung, Tanjung Pandang |
| **22** | *L. ingeri* | AB719242 | KUHE53848 | Malaysia, Sarawak, Matang |
| **23** | *L. kanowitense* | AB719255 | KUHE42590 | Malaysia, Sarawak, Kanowit |
| **24** | *L. kantonishikawai* | AB646391 | KUHE53562 | Malaysia, Sarawak, Bario |
| **25** | *L. leishanense* | EF544200 | IZCASH30004 | China, Guizhou Prov., Leigongshan Mt. |
| **26** | *L. leucops* | HQ709356 | BLS11838 | Vietnam, Lam Dong Prov., Bidoup - Nui Ba N.P. |
| **27** | *L. liui liui* | EF544182 | IOZCAS28061 | China, Fujian Prov., Wuyishan Mt. |
| **28** | *L. liui yaoshanense* | AB530441 | KUHEUNLL54 | China, Guangxi Prov., Huaping |
| **29** | *L. lumadorum* | AB530410 | ABTC76306 | Philippines, Mindanao Prov., Davao |
| **30** | *L. mangyanorum* | GQ995553 | KU304401 | Philippines, Mindoro Prov., Sablayan, Siburan Mt. |
| **31** | *L. masatakasatoi* | AB530445 | KUHE34396 | Laos, Phupan Prov., Xamneua |
| **32** | *L. montanum* | AB646386 | KUHE53783 | Malaysia, Sarawak, Kubah, Serapi Mt. |
| **33** | *L. montanum* | AB646385 | KUHE42817 | Indonesia, Central Kalimantan, Lamandau, Belantikan |
| **34** | *L. montanum* | AB646374 | KUHE44536 | Indonesia, East Kalimantan, Kutai |
| **35** | *L. mouhoti* | EF672272 | FMNH261758 | Cambodia, Mondulkiri Prov., O'Reang |
| **36** | *L. ngoclinhense* | EF544228 | ROMFS39612 | Vietnam, Quang Nam Prov., Ngoc Linh Mt. |
| **37** | *L. nigrops* | AB719249 | KUHE15430 | Malaysia, Selangor, Kuala Lumpur |
| **38** | *L. promustache* | EF544240 | IOZCAS2904-1 | China, Yunnan Prov., Hekou Co., Daweishan Mt. |
| **39** | *L. pullum* | MH581082 | ROMFS39611 | Vietnam, Kon Tum Prov., Kon Plong |
| **40** | *L. rakhinense* | JX127250 | CAS222296 | Myanmar, Rakhine State, Gwa township |
| **41** | *L. rakhinense* | DQ283239 | CAS222293 | Myanmar, Rakhine State, Gwa township |
| **42** | *L. smithi* | AB530432 | KUHE19281 | Thailand, Loei Prov., Phu Luang |
| **43** | *L. smithi* | AB530433 | KUHE19282 | Thailand, Loei Prov., Phu Luang |
| **44** | *L. smithi* | AB530434 | KUHE19834 | Thailand, Mae Hong Son Prov., Phasua W.F. |
| **45** | *L. smithi* | AB530435 | KUHE19839 | Thailand, Mae Hong Son Prov., Phasua W.F. |
| **46** | *L. smithi* | AB530436 | KUHE20200 | Thailand, Phetchaburi Prov., Kaeng Krachan |
| **47** | *L. smithi* | AB530437 | KUHE20201 | Thailand, Phetchaburi Prov., Kaeng Krachan |
| **48** | *L. smithi* | AB530438 | KUHE23342 | Thailand, Trang Prov., Kaochong |
| **49** | *L. smithi* | AB530439 | UMD0139 | Malaysia, Perlis, Langkawi |
| **50** | *L. smithi* | AB719246 | KUHE23318 | Thailand, Trang Prov., Kaochong |
| **51** | *L. smithi* | EF672271 | CAS222215 | Myanmar, Mon State, Kyaihto Township, Kyaik Hti Yo W.S. |
| **52** | *L. smithi* | GQ995541 | FMNH258092 | Laos, Sayaboury Prov. |
| **53** | *L. smithi* | GQ995542 | KUDSM910 | Thailand, no detailed locality data |
| **54** | *L. smithi* | JN848340 | MNHNP925 | Thailand, Phang Nga Prov. |
| **55** | *L. smithi* | JN848341 | MNHNP926 | Thailand, Phang Nga Prov. |
| **56** | *L. smithi* | JN848342 | MNHNP321 | Thailand, Phang Nga Prov. |
| **57** | *L. smithi* | JN848343 | MNHNP981 | Thailand, Phang Nga Prov. |
| **58** | *L. smithi* | JN848344 | MNHNK3182 | Laos, Luang Prabang Prov., Ban Sop Khao |
| **59** | *L. smithi* | JN848345 | MNHN2006.2424 | Laos, Luang Prabang Prov., Ban Sop Khao |
| **60** | *L. smithi* | JN848346 | MNHN2006.2431 | Laos, Luang Prabang Prov., Ban Keng Koung |
| **61** | *L. smithi* | JN848347 | MNHNK3266 | Laos, Luang Prabang Prov., Ban Van Thong |
| **62** | *L. smithi* | JN848349 | MNHN2006.2555-1 | Laos, Luang Prabang Prov., Luang Prabang |
| **63** | *L. smithi* | JN848350 | MNHNK3267 | Laos, Luang Prabang Prov., Ban Van Thong |
| **64** | *L. smithi* | JN848352 | LEL80 | Thailand, Chiang Mai Prov., Doi Chiang Dao Mt. |
| **65** | *L. smithi* | KR827850 | 20008227 | Thailand, Phang Nga Prov., Namtok Raman Forest Park |
| **66** | *L. smithi* | KR827851 | 0186Y | Thailand, Phetchabun Prov., Thung Salaeng Luang N.P. |
| **67** | *L. smithi* | KR827852 | 0158Y | Thailand, Phetchabun Prov., Thung Salaeng Luang N.P. |
| **68** | *L. smithi* | KR827853 | TADP981 | Thailand, Phang Nga Prov., Namtok Raman Forest Park |
| **69** | *L. smithi* | KR827854 | TADP925 | Thailand, Phang Nga Prov., Namtok Raman Forest Park |
| **70** | *L. smithi* | KR827855 | MNHNK3094 | Laos, Luang Prabang Prov., Ban Sop Khao |
| **71** | *L. smithi* | KR827856 | MNHN2006.2303 | Laos, Luang Prabang Prov., Ban Keng Koung |
| **72** | *L. smithi* | KR827857 | MNHN2006.2556 | Laos, Luang Prabang Prov., Houey Thao |
| **73** | *L. smithi* | KR827858 | MNHN2006.2555-2 | Laos, Luang Prabang Prov., Luang Prabang |
| **74** | *L. tagbanorum* | GQ995551 | PNMRMB3025 | Philippines, Palawan Prov., Nara |
| **75** | ***L. tenasserimense* sp. nov.** | AB530440 | KUHEUNAS2 | Thailand, Kanchanaburi Prov., Pilok Distr. |
| **76** | ***L. tenasserimense* sp. nov.** | MH581080 | ZMMU A-5918 | Thailand, Ratchaburi Prov., Suan Phung Distr. |
| **77** | ***L. tenasserimense* sp. nov.** | MH581081 | AUP-01284 | Thailand, Ratchaburi Prov., Suan Phung Distr. |
| **78** | *L. tengchongense* | KX066880 | SYSa004604d | China, Yunnan Prov., Tengchong |
| **79** | *L. waysepuntiense* | AB646388 | MZBAmp11313 | Indonesia, Sumatra, North Sumatra, Martabe |
| **80** | *L. xanthops* | JN711502 | NCSM78468 | Laos, Xe Kong Prov., Dakchung |
| **81** | *L. xanthospilum* | EF544231 | ROM32186 | Vietnam, Gia Lia Prov., Tram Lap |
|  | **Outgroup** |  |  |  |
|  | *Leptobrachella melanoleuca* | LC202000 | KUHE35710 | Thailand |
|  | *Megophrys nasuta* | LC202013 | KUHE53577 | Malaysia |
|  | *Oreolalax rhodostigmatus* | EF397248 | CIB-ZYCA746 | China, Guizhou Prov., Da Fang Co. |
|  | *Scutiger chintingensis* | EF397269 | ROM39065 | China, Sichuan Prov., Hongya Co. |
|  | *Pelodytes punctatus* | DQ283111 | no voucher | Spain, Catalonia, Barcelona |
