# Supplementary material for: A new species of Leptobrachium (Anura, Megophryidae) from western Thailand
Source: PeerJ. 2018 Aug 31;6:e5584. doi: 10.7717/peerj.5584 (PMC6120444; doi:10.7717/peerj.5584)
Supplement: Table S2 — Uncorrected p-distances (percentage) for 16S rRNA sequences (below diagonal) and calculation error (above diagonal) of the examined species of Leptobrachium. Within-clade genetic distances for are shown on the diagonal in bold. Continued on the next page. [file peerj-06-5584-s002.docx]

**SUPPLEMENTARY TABLE 2.**

|  | **Species** | **1** | **2** | **3** | **4** | **5** | **6** | **7** | **8** | **9** | **10** | **11** | **12** | **13** | **14** | **15** | **16** | **17** | **18** | **19** | **20** | **21** | **22** |
| --- | --- | --- | --- | --- | --- | --- | --- | --- | --- | --- | --- | --- | --- | --- | --- | --- | --- | --- | --- | --- | --- | --- | --- |
| **1** | *L. tenasserimense* **sp. nov.** | **0.8** | 1.4 | 1.7 | 2.0 | 2.0 | 2.0 | 2.0 | 2.1 | 2.2 | 2.2 | 2.1 | 2.0 | 2.1 | 2.0 | 2.0 | 1.8 | 2.2 | 2.0 | 2.3 | 2.3 | 2.2 | 2.4 |
| **2** | *L. smithi* | 10.4 | **1.3** | 1.1 | 1.9 | 1.9 | 1.7 | 1.8 | 1.9 | 1.9 | 1.9 | 1.8 | 1.8 | 1.9 | 1.7 | 1.9 | 2.0 | 2.1 | 1.9 | 1.8 | 2.2 | 1.8 | 1.9 |
| **3** | *L. rakhinense* | 10.5 | 7.0 | **0.0** | 2.0 | 2.1 | 2.0 | 1.9 | 2.0 | 1.9 | 1.9 | 2.0 | 2.0 | 2.1 | 1.9 | 2.0 | 2.0 | 2.3 | 1.9 | 2.1 | 2.2 | 1.9 | 2.2 |
| **4** | *L. montanum* | 15.1 | 15.3 | 14.5 | **2.3** | 1.4 | 1.3 | 0.6 | 1.2 | 1.3 | 1.0 | 1.4 | 1.4 | 1.4 | 1.8 | 1.7 | 1.9 | 2.0 | 1.8 | 1.8 | 1.8 | 1.9 | 1.9 |
| **5** | *L.* cf. *montanum* 1 | 14.1 | 14.5 | 14.6 | 7.2 | – | 1.2 | 1.4 | 1.5 | 1.4 | 1.3 | 1.6 | 1.6 | 1.7 | 1.9 | 1.6 | 2.1 | 2.0 | 2.0 | 2.0 | 1.9 | 2.0 | 2.1 |
| **6** | *L.* cf. *montanum* 2 | 14.8 | 13.8 | 13.5 | 8.2 | 5.8 | – | 1.4 | 1.4 | 1.3 | 1.2 | 1.6 | 1.5 | 1.6 | 1.7 | 1.6 | 1.8 | 1.9 | 1.8 | 2.0 | 2.0 | 2.1 | 2.1 |
| **7** | *L. waysepuntiense* | 15.4 | 15.2 | 14.4 | 1.9 | 7.3 | 7.7 | – | 1.2 | 1.2 | 1.1 | 1.4 | 1.4 | 1.4 | 1.7 | 1.8 | 1.9 | 2.0 | 1.7 | 1.8 | 1.8 | 1.9 | 1.8 |
| **8** | *L. kantonishikawai* | 15.3 | 15.1 | 14.3 | 6.3 | 8.5 | 8.6 | 5.5 | – | 1.2 | 1.3 | 1.4 | 1.4 | 1.5 | 1.7 | 1.8 | 2.2 | 2.1 | 1.9 | 1.9 | 2.0 | 1.9 | 2.0 |
| **9** | *L. gunungense* | 15.7 | 15.6 | 14.3 | 5.5 | 7.9 | 7.3 | 4.6 | 6.4 | – | 0.9 | 1.2 | 1.5 | 1.3 | 1.6 | 1.7 | 1.9 | 1.9 | 1.7 | 1.9 | 1.9 | 2.0 | 2.0 |
| **10** | *L. abbotti* | 15.7 | 15.7 | 14.9 | 4.0 | 6.1 | 8.0 | 3.7 | 6.4 | 3.4 | – | 1.3 | 1.3 | 1.5 | 1.7 | 1.7 | 1.9 | 1.9 | 1.7 | 1.8 | 1.9 | 1.9 | 2.0 |
| **11** | *L. tagbanorum* | 16.7 | 16.2 | 15.1 | 8.8 | 9.8 | 9.9 | 8.3 | 9.8 | 7.1 | 7.7 | – | 1.6 | 1.4 | 1.8 | 1.7 | 1.9 | 1.9 | 1.6 | 1.7 | 1.8 | 1.8 | 1.8 |
| **12** | *L. lumadorum* | 16.9 | 16.1 | 16.8 | 9.3 | 10.7 | 8.9 | 8.6 | 10.1 | 9.5 | 9.2 | 12.6 | – | 1.6 | 1.8 | 1.8 | 2.1 | 2.0 | 1.9 | 2.0 | 2.2 | 2.0 | 2.0 |
| **13** | *L. mangyanorum* | 13.7 | 15.2 | 14.6 | 9.2 | 10.5 | 10.6 | 9.0 | 9.3 | 7.4 | 8.7 | 7.1 | 11.8 | – | 1.9 | 1.8 | 2.1 | 1.9 | 1.8 | 2.0 | 1.8 | 1.9 | 2.0 |
| **14** | *L. hasseltii* | 15.3 | 13.6 | 13.8 | 11.8 | 10.7 | 11.4 | 11.7 | 11.9 | 11.3 | 10.7 | 13.9 | 13.5 | 15.2 | – | 1.6 | 1.8 | 1.7 | 1.9 | 1.9 | 1.7 | 1.9 | 1.8 |
| **15** | *L. hendricksoni* | 17.2 | 17.1 | 15.6 | 15.4 | 13.2 | 15.4 | 16.2 | 15.5 | 16.1 | 15.2 | 16.7 | 18.5 | 17.4 | 9.8 | **3.1** | 2.0 | 2.0 | 1.8 | 1.6 | 1.8 | 1.6 | 1.7 |
| **16** | *L. nigrops* | 15.2 | 19.1 | 16.5 | 14.6 | 16.2 | 16.2 | 15.3 | 16.8 | 15.2 | 14.9 | 17.8 | 17.1 | 17.6 | 11.3 | 14.4 | – | 1.6 | 1.7 | 2.1 | 2.0 | 2.0 | 2.1 |
| **17** | *L. ingeri* | 16.9 | 18.1 | 16.9 | 14.2 | 14.7 | 15.5 | 14.7 | 15.7 | 15.0 | 14.4 | 16.2 | 16.1 | 15.8 | 9.6 | 14.7 | 8.9 | **4.6** | 1.7 | 1.9 | 1.9 | 1.9 | 2.0 |
| **18** | *L. kanowitense* | 18.2 | 19.5 | 17.3 | 13.0 | 16.4 | 16.1 | 12.7 | 15.1 | 14.8 | 13.6 | 14.6 | 17.3 | 16.6 | 13.0 | 15.5 | 11.7 | 11.4 | – | 1.9 | 1.9 | 1.9 | 2.0 |
| **19** | *L. chapaense* | 20.5 | 17.5 | 17.7 | 17.8 | 15.9 | 17.1 | 17.7 | 19.2 | 16.8 | 17.1 | 17.2 | 18.3 | 18.3 | 15.0 | 15.8 | 20.1 | 17.4 | 19.4 | – | 1.4 | 0.8 | 1.3 |
| **20** | *L.* cf. *chapaense* | 19.9 | 16.7 | 17.7 | 16.5 | 15.5 | 15.6 | 16.5 | 18.9 | 15.5 | 16.5 | 17.2 | 18.0 | 17.3 | 15.3 | 16.6 | 20.7 | 18.5 | 19.1 | 9.8 | – | 1.5 | 1.5 |
| **21** | *L. huashen* | 21.2 | 18.6 | 17.9 | 18.8 | 16.8 | 17.2 | 18.9 | 19.7 | 17.7 | 18.2 | 18.8 | 19.8 | 19.7 | 16.0 | 16.7 | 20.8 | 19.0 | 20.3 | 2.5 | 10.5 | **1.4** | 1.2 |
| **22** | *L. ailaonicum* | 20.6 | 18.4 | 19.4 | 17.2 | 17.3 | 18.2 | 17.3 | 19.2 | 17.3 | 17.9 | 17.1 | 19.0 | 18.1 | 15.2 | 17.5 | 20.6 | 18.6 | 20.1 | 6.1 | 10.1 | 6.9 | **2.4** |

**SUPPLEMENTARY TABLE 2.** Continued.

|  | **Species** | **23** | **24** | **25** | **26** | **27** | **28** | **29** | **30** | **31** | **32** | **33** | **34** | **35** | **36** | **37** | **38** |
| --- | --- | --- | --- | --- | --- | --- | --- | --- | --- | --- | --- | --- | --- | --- | --- | --- | --- |
| **1** | *L. tenasserimense* **sp. nov.** | 2.4 | 2.3 | 2.5 | 2.4 | 2.2 | 2.4 | 2.0 | 2.2 | 2.5 | 2.5 | 2.2 | 2.3 | 2.3 | 2.4 | 2.4 | 2.3 |
| **2** | *L. smithi* | 2.0 | 2.0 | 2.1 | 2.1 | 1.8 | 2.0 | 1.8 | 1.9 | 2.2 | 2.2 | 2.1 | 2.0 | 2.1 | 2.0 | 2.0 | 2.2 |
| **3** | *L. rakhinense* | 2.1 | 2.1 | 2.2 | 2.2 | 2.1 | 2.3 | 2.0 | 2.1 | 2.3 | 2.4 | 2.3 | 2.2 | 2.2 | 2.2 | 2.1 | 2.2 |
| **4** | *L. montanum* | 1.7 | 1.7 | 2.0 | 2.0 | 1.9 | 2.0 | 2.0 | 1.8 | 2.0 | 2.1 | 1.9 | 1.8 | 1.8 | 1.9 | 1.8 | 1.9 |
| **5** | *L.* cf. *montanum* 1 | 2.0 | 2.0 | 2.2 | 2.1 | 2.2 | 2.2 | 2.2 | 2.0 | 2.2 | 2.3 | 2.0 | 2.1 | 2.1 | 2.1 | 2.0 | 2.2 |
| **6** | *L.* cf. *montanum* 2 | 2.0 | 2.0 | 2.1 | 2.1 | 2.0 | 2.2 | 2.0 | 1.8 | 2.1 | 2.2 | 1.9 | 2.0 | 2.0 | 2.0 | 2.0 | 2.0 |
| **7** | *L. waysepuntiense* | 1.7 | 1.7 | 2.0 | 2.0 | 2.0 | 1.9 | 2.0 | 1.8 | 2.0 | 2.1 | 1.8 | 1.9 | 1.8 | 1.8 | 1.8 | 1.9 |
| **8** | *L. kantonishikawai* | 1.8 | 1.8 | 2.0 | 2.0 | 2.1 | 2.1 | 2.0 | 2.0 | 2.1 | 2.2 | 2.0 | 1.9 | 1.9 | 2.0 | 2.0 | 2.0 |
| **9** | *L. gunungense* | 1.9 | 1.8 | 2.1 | 2.0 | 2.0 | 2.1 | 1.9 | 1.8 | 2.0 | 2.1 | 1.8 | 1.8 | 1.8 | 1.9 | 1.9 | 1.9 |
| **10** | *L. abbotti* | 1.8 | 1.8 | 2.0 | 2.0 | 2.1 | 2.1 | 1.9 | 1.8 | 2.0 | 2.1 | 1.9 | 1.9 | 1.8 | 1.9 | 1.9 | 1.9 |
| **11** | *L. tagbanorum* | 1.7 | 1.7 | 2.1 | 2.0 | 1.9 | 2.1 | 2.0 | 1.8 | 1.9 | 1.9 | 1.8 | 1.8 | 1.8 | 1.9 | 1.8 | 1.9 |
| **12** | *L. lumadorum* | 1.9 | 2.0 | 2.1 | 2.1 | 2.1 | 2.2 | 1.8 | 1.8 | 2.1 | 2.2 | 2.1 | 2.0 | 1.9 | 2.0 | 1.8 | 1.9 |
| **13** | *L. mangyanorum* | 1.9 | 1.8 | 2.2 | 2.1 | 2.1 | 2.2 | 2.0 | 2.0 | 2.0 | 2.0 | 1.9 | 1.9 | 1.8 | 1.9 | 2.0 | 1.8 |
| **14** | *L. hasseltii* | 1.7 | 1.7 | 1.8 | 1.9 | 1.9 | 1.9 | 2.0 | 1.9 | 2.0 | 2.1 | 2.0 | 1.9 | 2.0 | 2.0 | 2.0 | 2.0 |
| **15** | *L. hendricksoni* | 1.6 | 1.7 | 1.7 | 1.7 | 1.8 | 1.9 | 1.9 | 1.9 | 1.8 | 1.9 | 2.0 | 2.1 | 1.9 | 2.0 | 1.9 | 1.9 |
| **16** | *L. nigrops* | 2.1 | 2.0 | 2.1 | 2.2 | 2.1 | 1.9 | 1.9 | 2.0 | 2.1 | 2.2 | 1.8 | 1.9 | 1.9 | 2.0 | 2.1 | 1.9 |
| **17** | *L. ingeri* | 2.0 | 1.9 | 2.0 | 2.0 | 1.9 | 2.1 | 1.7 | 2.0 | 2.2 | 2.3 | 2.0 | 1.9 | 2.1 | 2.1 | 2.1 | 2.2 |
| **18** | *L. kanowitense* | 1.9 | 1.9 | 1.9 | 2.0 | 1.9 | 2.0 | 2.0 | 1.9 | 1.9 | 2.0 | 1.9 | 1.9 | 1.7 | 1.8 | 1.9 | 1.7 |
| **19** | *L. chapaense* | 1.1 | 1.1 | 1.3 | 1.3 | 1.2 | 1.5 | 1.8 | 1.9 | 1.9 | 1.9 | 1.9 | 2.0 | 1.8 | 1.8 | 1.8 | 1.8 |
| **20** | *L.* cf. *chapaense* | 1.5 | 1.5 | 1.5 | 1.6 | 1.4 | 1.7 | 2.0 | 2.2 | 2.2 | 2.2 | 2.0 | 2.4 | 2.2 | 2.2 | 2.2 | 2.1 |
| **21** | *L. huashen* | 1.0 | 1.0 | 1.3 | 1.3 | 1.2 | 1.4 | 1.8 | 1.8 | 2.0 | 2.0 | 1.9 | 2.0 | 1.9 | 1.9 | 1.8 | 1.8 |
| **22** | *L. ailaonicum* | 1.0 | 1.0 | 1.2 | 1.4 | 1.1 | 1.1 | 2.0 | 1.9 | 2.1 | 1.9 | 2.0 | 2.1 | 1.9 | 2.0 | 1.9 | 1.9 |

**SUPPLEMENTARY TABLE 2.** Continued.

|  | **Species** | **1** | **2** | **3** | **4** | **5** | **6** | **7** | **8** | **9** | **10** | **11** | **12** | **13** | **14** | **15** | **16** | **17** | **18** | **19** | **20** | **21** | **22** |
| --- | --- | --- | --- | --- | --- | --- | --- | --- | --- | --- | --- | --- | --- | --- | --- | --- | --- | --- | --- | --- | --- | --- | --- |
| **23** | *L. liui* | 22.0 | 18.9 | 18.3 | 18.4 | 17.4 | 18.0 | 18.5 | 20.7 | 18.0 | 18.6 | 18.8 | 19.4 | 19.5 | 16.5 | 17.8 | 21.8 | 19.8 | 21.8 | 5.6 | 9.8 | 6.3 | 4.7 |
| **24** | *L. leishanense* | 22.1 | 18.8 | 18.3 | 17.9 | 17.4 | 18.0 | 18.0 | 20.4 | 17.4 | 18.3 | 18.5 | 19.6 | 19.2 | 16.8 | 18.4 | 21.3 | 20.0 | 21.3 | 5.8 | 8.5 | 5.8 | 4.4 |
| **25** | *L. boringii* | 22.7 | 20.0 | 18.9 | 19.8 | 18.6 | 19.0 | 20.5 | 21.3 | 19.5 | 19.8 | 20.6 | 21.4 | 21.4 | 16.5 | 17.5 | 20.4 | 20.0 | 19.8 | 7.0 | 11.0 | 7.4 | 7.0 |
| **26** | *L. masatakasatoi* | 21.4 | 19.3 | 19.5 | 18.8 | 17.4 | 18.3 | 18.7 | 19.5 | 18.0 | 18.6 | 19.1 | 19.3 | 20.1 | 15.9 | 18.1 | 22.0 | 19.4 | 21.3 | 6.4 | 11.6 | 6.8 | 7.3 |
| **27** | *L. tengchongense* | 20.2 | 17.7 | 18.3 | 17.3 | 16.2 | 17.7 | 17.4 | 19.5 | 17.4 | 17.7 | 17.5 | 19.6 | 18.6 | 15.6 | 16.9 | 20.1 | 17.9 | 20.1 | 5.5 | 9.8 | 6.3 | 4.4 |
| **28** | *L. promustache* | 23.6 | 20.7 | 21.3 | 20.1 | 20.7 | 21.1 | 20.2 | 22.6 | 19.8 | 21.3 | 20.0 | 22.0 | 21.1 | 18.3 | 19.6 | 21.3 | 20.9 | 20.7 | 9.1 | 11.6 | 9.7 | 6.4 |
| **29** | *L. banae* | 20.2 | 18.1 | 19.2 | 19.1 | 18.9 | 19.3 | 18.7 | 20.1 | 19.8 | 19.5 | 20.3 | 19.0 | 20.1 | 18.0 | 19.3 | 20.4 | 18.6 | 17.9 | 16.2 | 17.7 | 17.5 | 17.6 |
| **30** | *L. xanthospilum* | 20.2 | 16.3 | 19.2 | 15.2 | 15.9 | 16.2 | 15.6 | 16.5 | 15.9 | 15.2 | 18.2 | 15.9 | 18.6 | 14.1 | 17.2 | 17.4 | 16.5 | 17.3 | 14.6 | 15.9 | 16.4 | 15.4 |
| **31** | *L. guangxiense* | 22.1 | 19.3 | 21.3 | 18.2 | 18.0 | 18.7 | 18.7 | 19.5 | 17.7 | 18.3 | 18.8 | 19.6 | 20.7 | 16.8 | 18.7 | 18.9 | 18.9 | 19.4 | 16.2 | 17.7 | 17.7 | 17.3 |
| **32** | *L. hainanense* | 22.1 | 19.3 | 21.6 | 18.2 | 18.3 | 18.7 | 18.7 | 19.5 | 18.3 | 18.9 | 18.5 | 19.6 | 20.7 | 17.4 | 19.0 | 19.5 | 19.5 | 19.4 | 16.8 | 17.7 | 18.3 | 16.9 |
| **33** | *L. leucops* | 19.5 | 17.1 | 18.8 | 16.2 | 15.4 | 17.0 | 16.4 | 17.3 | 17.0 | 17.0 | 18.7 | 17.6 | 19.4 | 14.6 | 16.9 | 17.0 | 16.8 | 16.9 | 13.6 | 14.5 | 15.0 | 15.1 |
| **34** | *L. buchardi* | 21.4 | 18.5 | 21.0 | 16.7 | 17.1 | 18.3 | 16.5 | 18.0 | 17.7 | 17.4 | 19.7 | 18.7 | 20.7 | 15.6 | 19.3 | 18.3 | 17.3 | 18.2 | 15.9 | 17.4 | 17.6 | 16.8 |
| **35** | *L. xanthops* | 19.1 | 17.3 | 19.6 | 15.6 | 16.2 | 16.9 | 15.3 | 16.8 | 16.5 | 16.2 | 18.2 | 17.2 | 18.6 | 16.0 | 18.5 | 18.0 | 18.2 | 16.4 | 14.7 | 16.2 | 16.4 | 15.2 |
| **36** | *L. ngoclinhense* | 20.2 | 17.8 | 20.1 | 16.0 | 15.9 | 17.1 | 15.9 | 17.4 | 16.8 | 16.5 | 18.5 | 17.1 | 18.9 | 15.9 | 18.7 | 18.3 | 18.3 | 17.6 | 14.6 | 16.2 | 16.4 | 15.7 |
| **37** | *L. pullum* | 21.7 | 18.1 | 20.1 | 17.4 | 18.0 | 19.0 | 17.7 | 19.2 | 18.6 | 18.3 | 19.7 | 17.4 | 20.1 | 17.7 | 20.2 | 19.5 | 19.7 | 18.8 | 15.2 | 16.5 | 17.0 | 16.2 |
| **38** | *L. mouhoti* | 20.2 | 18.4 | 20.1 | 16.8 | 17.4 | 18.7 | 17.1 | 18.3 | 17.4 | 17.1 | 19.1 | 17.1 | 18.9 | 16.5 | 18.7 | 18.0 | 18.3 | 17.0 | 14.6 | 16.2 | 16.4 | 16.2 |

**SUPPLEMENTARY TABLE 2.** Continued.

|  | **Species** | **23** | **24** | **25** | **26** | **27** | **28** | **29** | **30** | **31** | **32** | **33** | **34** | **35** | **36** | **37** | **38** |
| --- | --- | --- | --- | --- | --- | --- | --- | --- | --- | --- | --- | --- | --- | --- | --- | --- | --- |
| **23** | *L. liui* | **2.7** | 0.8 | 1.1 | 1.3 | 1.1 | 1.2 | 2.1 | 1.9 | 2.1 | 2.1 | 2.0 | 2.2 | 1.9 | 2.0 | 1.9 | 1.9 |
| **24** | *L. leishanense* | 3.2 | – | 1.1 | 1.3 | 1.1 | 1.3 | 2.2 | 2.0 | 2.1 | 2.2 | 2.1 | 2.3 | 2.0 | 2.1 | 2.0 | 2.0 |
| **25** | *L. boringii* | 5.8 | 5.5 | – | 1.2 | 1.3 | 1.3 | 2.0 | 2.0 | 2.0 | 2.1 | 2.1 | 2.2 | 2.1 | 2.1 | 2.1 | 2.1 |
| **26** | *L. masatakasatoi* | 7.0 | 7.0 | 7.0 | – | 1.4 | 1.5 | 1.9 | 1.9 | 2.1 | 2.2 | 1.9 | 2.2 | 1.9 | 1.9 | 2.0 | 2.0 |
| **27** | *L. tengchongense* | 4.4 | 4.3 | 7.0 | 6.4 | – | 1.2 | 1.9 | 1.8 | 2.0 | 2.0 | 1.8 | 2.0 | 1.9 | 1.9 | 1.8 | 1.8 |
| **28** | *L. promustache* | 7.8 | 7.0 | 8.5 | 10.4 | 7.0 | – | 2.2 | 2.1 | 2.2 | 2.3 | 2.2 | 2.3 | 2.2 | 2.2 | 2.1 | 2.1 |
| **29** | *L. banae* | 17.8 | 17.1 | 16.8 | 16.8 | 17.4 | 18.3 | – | 1.5 | 1.6 | 1.6 | 1.6 | 1.6 | 1.5 | 1.4 | 1.6 | 1.6 |
| **30** | *L. xanthospilum* | 16.5 | 15.9 | 15.5 | 15.2 | 14.9 | 16.2 | 9.8 | – | 1.5 | 1.7 | 1.5 | 1.4 | 1.3 | 1.2 | 1.5 | 1.5 |
| **31** | *L. guangxiense* | 17.8 | 18.3 | 17.4 | 16.5 | 16.2 | 19.2 | 11.3 | 8.8 | **0.0** | 0.5 | 1.5 | 1.3 | 1.4 | 1.2 | 1.4 | 1.3 |
| **32** | *L. hainanense* | 18.4 | 18.3 | 17.4 | 17.1 | 16.8 | 18.9 | 11.0 | 9.1 | 0.9 | – | 1.6 | 1.4 | 1.3 | 1.3 | 1.5 | 1.4 |
| **33** | *L. leucops* | 15.9 | 15.7 | 14.8 | 14.5 | 14.2 | 16.7 | 11.1 | 7.7 | 7.4 | 7.4 | – | 1.1 | 1.4 | 1.2 | 1.4 | 1.4 |
| **34** | *L. buchardi* | 18.1 | 18.0 | 17.7 | 17.4 | 16.5 | 18.3 | 11.6 | 7.3 | 7.0 | 7.3 | 5.2 | – | 1.4 | 1.2 | 1.3 | 1.3 |
| **35** | *L. xanthops* | 16.1 | 16.2 | 15.6 | 15.3 | 15.3 | 17.1 | 10.7 | 7.3 | 6.7 | 6.4 | 6.2 | 6.4 | – | 1.0 | 1.1 | 1.1 |
| **36** | *L. ngoclinhense* | 16.3 | 15.5 | 15.9 | 14.9 | 15.2 | 17.7 | 9.5 | 5.2 | 4.9 | 5.2 | 5.2 | 4.3 | 4.0 | – | 1.0 | 0.9 |
| **37** | *L. pullum* | 16.9 | 16.8 | 16.5 | 16.5 | 15.5 | 16.8 | 11.9 | 7.3 | 7.6 | 7.3 | 6.5 | 6.7 | 5.8 | 4.6 | – | 1.0 |
| **38** | *L. mouhoti* | 16.9 | 16.8 | 16.5 | 16.2 | 15.2 | 17.7 | 11.0 | 7.0 | 6.1 | 6.4 | 5.9 | 5.8 | 4.3 | 3.0 | 3.4 | – |
